# Jejunal Intussusception Due to Heterotopic Pancreas: A Case Report

**DOI:** 10.7759/cureus.14586

**Published:** 2021-04-20

**Authors:** Nsikak E Daniel, Fidel S Rampersad, Vijay Naraynsingh, Shaheeba Barrow, Stephan David

**Affiliations:** 1 Radiology, Eric Williams Medical Sciences Complex, Trinidad, TTO; 2 Radiology, The University of the West Indies, Port of Spain, TTO; 3 Clinical Surgical Sciences, The University of the West Indies, Port of Spain, TTO; 4 Surgery, Medical Associates Hospital, St. Joseph, TTO; 5 Pathology, Port of Spain General Hospital, Port of Spain, TTO

**Keywords:** ectopic pancreas, jejunojejunal intussusception, intussusception in the elderly, laparotomy, risk of malignancy, pancreatic heterotopia, lead point, small bowel obstruction, target sign, abdominal pain

## Abstract

Intussusception in adults is rare. Even more unusual is jejunal intussusception secondary to a heterotopic pancreas. The presence of pancreatic tissue in an ectopic location and lacking contiguity with the main pancreatic gland is defined as pancreatic heterotopia. It is very rarely symptomatic and usually diagnosed incidentally during surgical intervention for other conditions. We report the case of a 78-year-old lady who presented with a history of constipation, abdominal pain, and vomiting. A CT scan revealed features of a proximal jejunojejunal intussusception secondary to a small soft tissue density lead point. After laparotomy and segmental jejunal resection, histopathology confirmed the diagnosis of ectopic pancreatic tissue as the lead point. Although uncommon, heterotopic pancreatic tissue should be included in the differential diagnosis for proximal small bowel intussusception.

## Introduction

Pancreatic heterotopia is rare and is defined as pancreatic tissue lacking anatomical and vascular continuity with the main pancreas [1]. The pancreatic deposits predominantly occur within the stomach, duodenum, and jejunum, and, to a lesser extent, the ileum. 

There are two leading theories surrounding the origin of pancreatic ectopia, with the first being referred to as the “poor position” theory and the second as the “metaplasia” theory [2-3]. The “poor position theory” proposes that during embryogenesis, elements of the primitive pancreas can be separated from each other during intestinal rotation and, consequently, develop within other tissues in varying locations. The “metaplasia theory” proposes that the heterotopic pancreas develops from the foci of pancreatic metaplasia, which subsequently migrate during embryogenesis to the ectopic position.

The ectopic pancreas is rarely diagnosed upon presentation, and preoperative diagnosis is unusual [4]. Ectopic pancreatic tissue is usually discovered incidentally and often generally asymptomatic unless complicated by inflammation (from the release of pancreatic enzymes), obstruction, gastrointestinal bleeding, or malignant transformation [5-6]. However, when they do present with symptoms, the most common presentation is usually abdominal pain [7]. An ectopic pancreas as the main cause of jejunal intussusception in adults is extremely rare. Herein, we describe such a case of jejunal ectopic pancreas causing jejunojejunal intussusception.

## Case presentation

**Figure 1:**
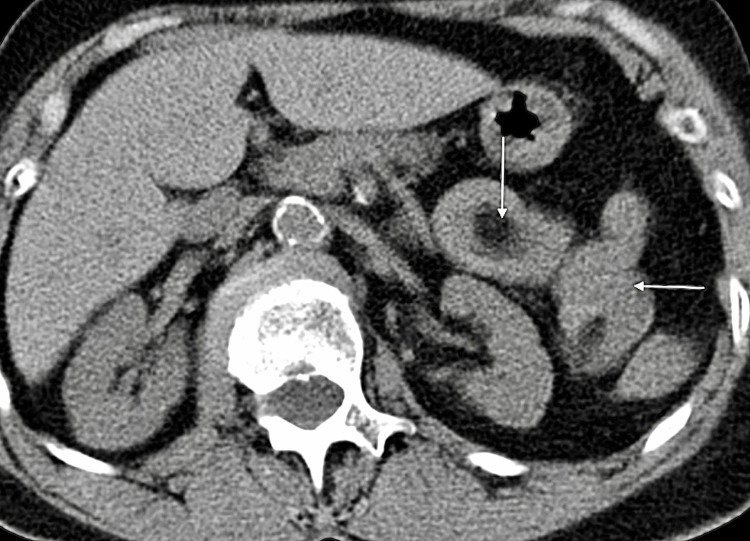
Axial non-contrast CT scan to the upper abdomen showing the “target sign” as evidenced by alternating varying mural densities within the proximal jejunum (vertical arrow) There is also focal mural thickening at the antimesenteric border of the jejunum (horizontal arrow).

**Figure 2:**
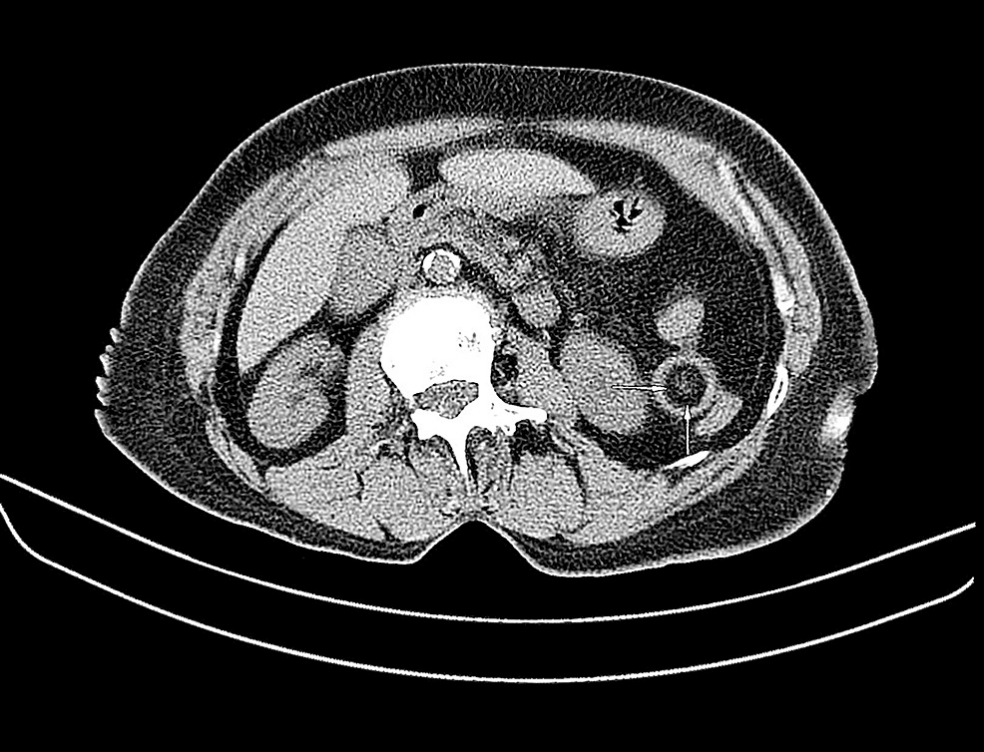
Axial non-contrast CT image through the upper abdomen demonstrating fat density (HU of - 85) within the lumen of the proximal jejunum (horizontal and vertical arrows), representing mesenteric fat

**Figure 3:**
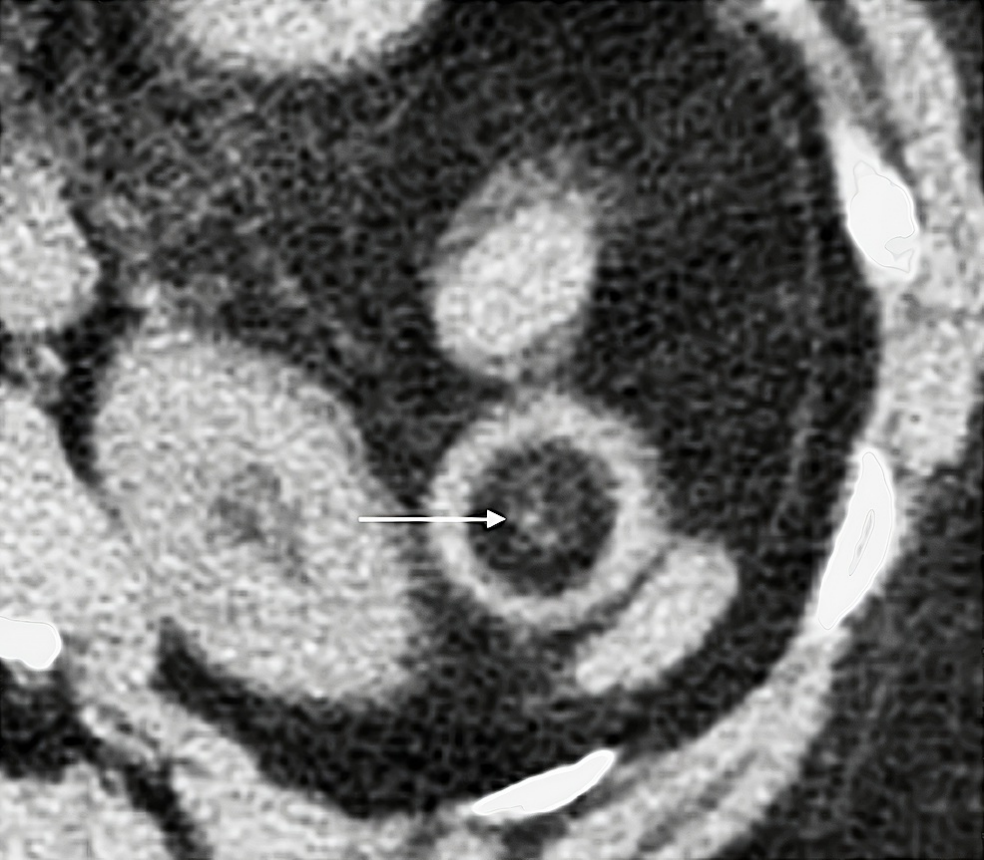
Axial CT through the jejunum (magnified) at the level of the left kidney shows jejunal intraluminal fat density, consistent with mesenteric fat (horizontal arrow)

**Figure 4:**
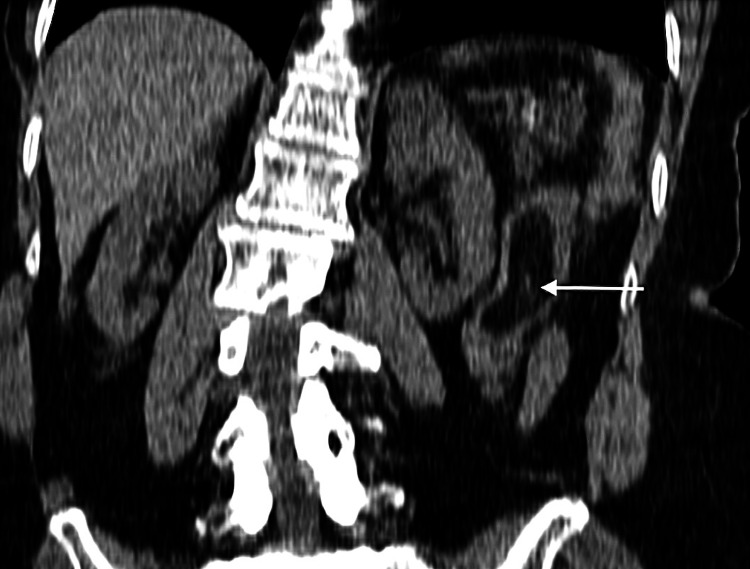
Reformatted coronal noncontrast CT through the upper abdomen showing intraluminal fat density within the jejunum (horizontal arrow)

**Figure 5:**
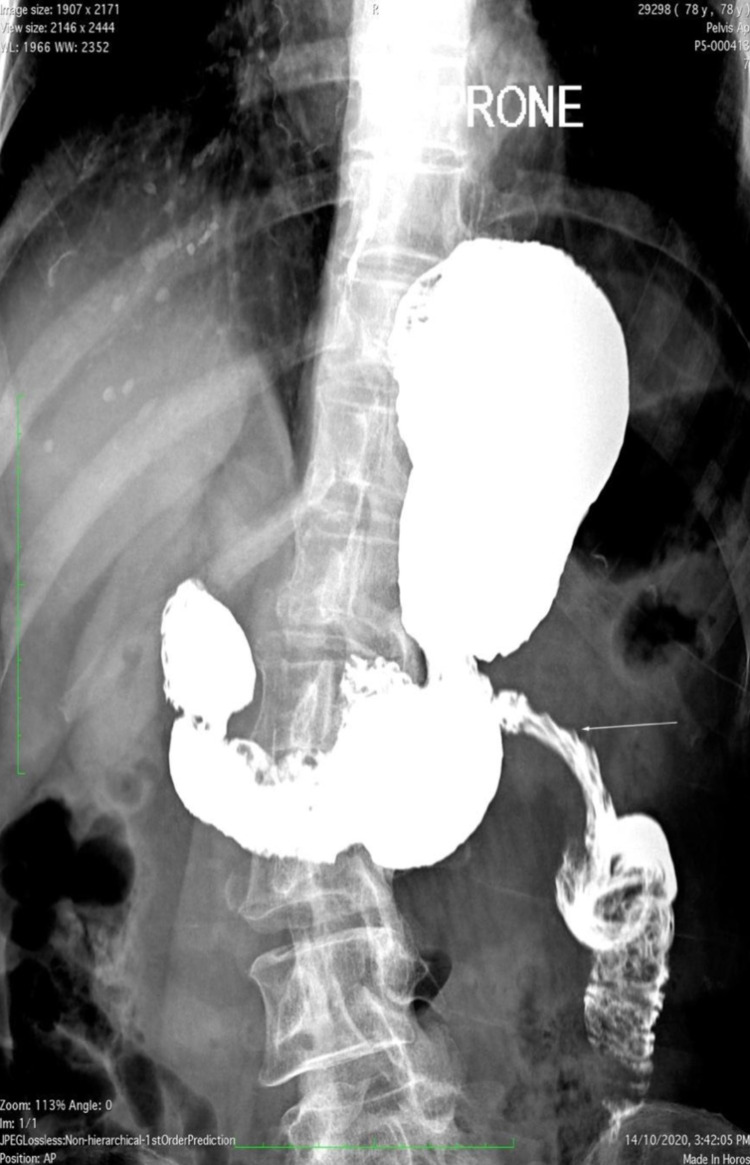
Prone view of a barium follow-through study demonstrating a focally narrowed segment of proximal jejunum at the area of the CT detected jejunal intussusception, consistent with reactive mural edema There is no tight stricture, with no evidence of a mass or filling defect.

A 78-year-old female of Indian descent presented with a one-week history of constipation, worsening abdominal pain, and vomiting for two days. Examination revealed a well-looking patient with normal vital signs. She had mild upper abdominal tenderness and guarding in the epigastrium and left upper quadrant. Laboratory tests, abdominal ultrasound, and abdominal X-rays were unremarkable, showing no bowel obstruction or mass lesions. Computed tomography (CT) scan of the abdomen and pelvis without intravenous (IV) contrast showed a short segment of jejunal mural thickening and alternate varying concentric jejunal mural densities (target sign) (Figure 1), consistent with a jejunojejunal intussusception, with intraluminal fat and a subcentimeter soft tissue density mural lesion (Figures 2-4), with the latter thought to be the lead point. The patient settled on conservative management and a laparotomy was planned. A barium meal and follow-through prior to surgery were performed two days after the initial CT, which showed a narrowing of the lumen in the proximal jejunum likely due to jejunal mural edema after spontaneous intussusception reduction (Figure 5). No filling defects were identified.

**Figure 6:**
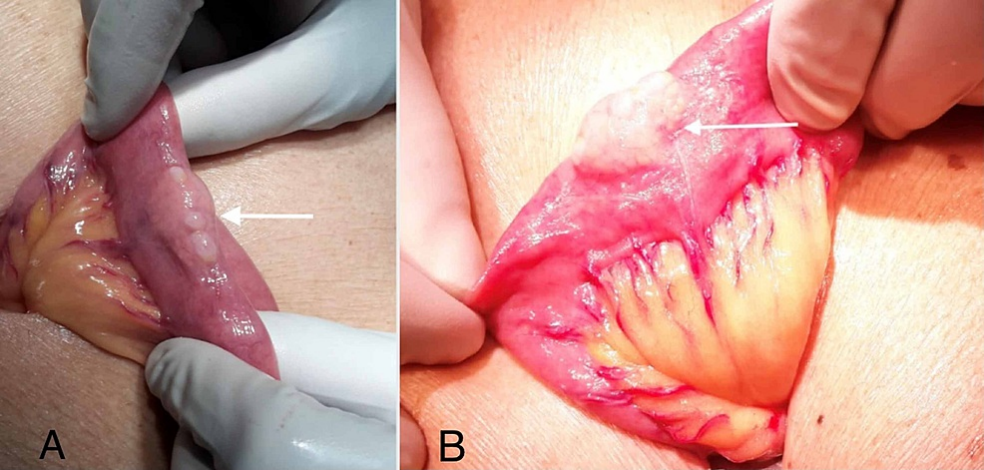
6a-6b: Intraoperative laparotomy photographs revealing a segment of mobilized jejunum with a mural mass (horizontal arrows) on the antimesenteric border of the jejunum

**Figure 7:**
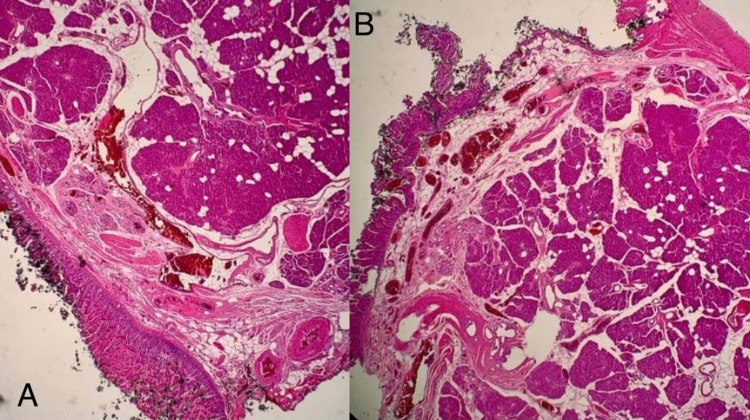
7a-7b: Histology micrographs at 20x magnification showing ectopic pancreatic tissue expanding the submucosa of the wall of the jejunum

**Figure 8:**
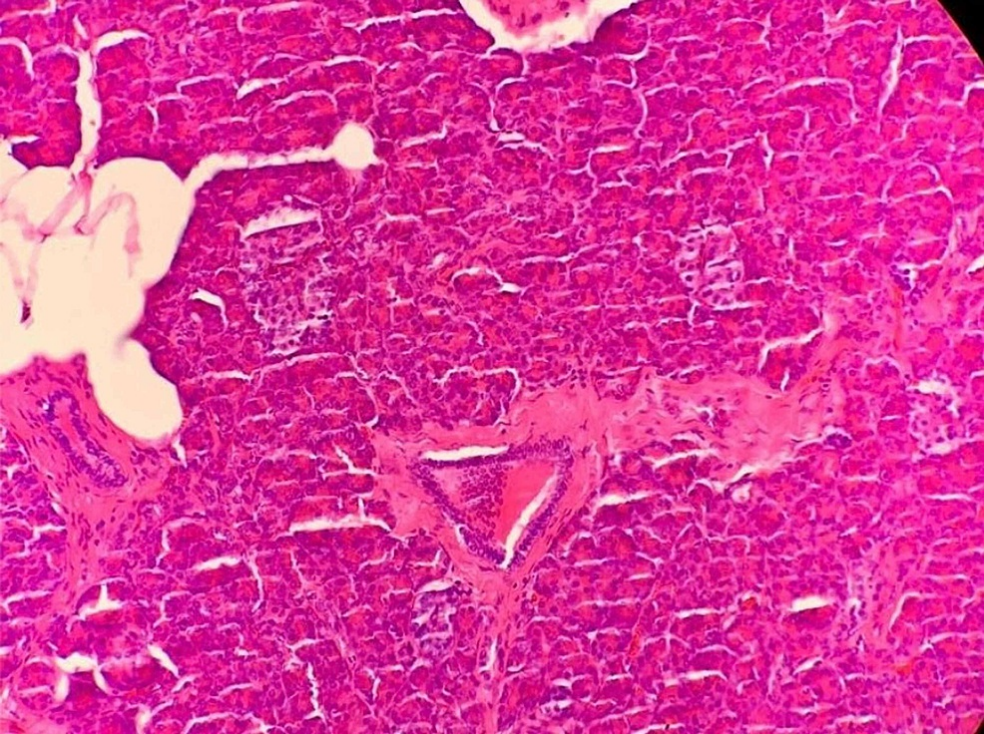
Histology micrographs at 200x magnification showing fully formed, mature heterotopic pancreatic tissue with acini and ducts, within the jejunal wall

The patient then underwent laparotomy and a small mass was detected on the antimesenteric border of the proximal jejunum (Figures 6a-6b). This segment of jejunum was resected, and a primary anastomosis was performed. Histopathology demonstrated ectopic pancreatic tissue expanding the submucosa of the bowel wall (Figures 7a-7b,8). The patient had an uneventful recovery and was discharged two days later.

## Discussion

Ectopic pancreatic tissue can be found in the subserosal, submucosal, or muscularis layers of various portions of the gastrointestinal tract. A focus of pancreatic tissue, which is not in continuity with the main pancreas, is referred to as an ectopic pancreas. It is also referred to as a heterotopic, aberrant, or accessory pancreas [8].

Approximately 75% of pancreatic heterotopia occurs in the stomach, primarily located within the gastric antrum. It has also been reported to occur at other sites, namely, the duodenum, jejunum, ileum, mediastinum, umbilicus, gallbladder, common bile duct, Meckel’s diverticulum, fallopian tubes, and the splenic hilum [1,9]. The first reported case of a heterotopic pancreas was reported to have developed in an ileal diverticulum by Jean Schultz in 1727.

The first histologically confirmed case was in the 19th century by Klob in 1859. According to the first histologic classification, which was described by Heinrich in 1909, there are three types of heterotopic pancreas. This classification was further modified by Gasper-Fuentes in 1973 to include a fourth type. The first and most common type, which also happens to be the type reported in our case, is composed of all the elements of a normal pancreas, which include pancreatic acini, islet cells, and ducts. The original classification describes the second and third types as being predominantly either pancreatic acini or ducts, with no islet cells. According to Gasper-Fuentes, however, the second and third types consist of only ducts and acini, respectively, while the fourth type is composed of only islet cells [10].

Due to the mostly asymptomatic nature of heterotopic pancreas, they are usually discovered incidentally during laparotomy or autopsy. They have an incidence of 0.2% at laparotomy and 0.5% to 3.7% on autopsy [11]. Upon incidental discovery of an ectopic pancreas, surgical resection is advised due to the risk of late clinical problems [4].

At least 50% of small bowel heterotopic pancreas are asymptomatic. When they do become symptomatic, the most frequent presentation is abdominal pain due to inflammation resulting from a release of pancreatic enzymes [9]. The clinical features could be as a result of hemorrhage into the ectopic pancreatic tissue with subsequent mucosal erosion and ulcer formation; this is most commonly seen in small bowel lesions [12-13]. With larger lesions, especially those greater than 1.5 cm, patients are more likely to have other symptoms such as nausea, vomiting, and gastrointestinal bleeding. Malignant transformation of heterotopic pancreas has also been reported [5].

It is extremely rare for a heterotopic pancreas to cause obstruction or intussusception. Such case reports reveal that the ectopic pancreas is usually located within an ileal Meckel’s diverticulum, which serves as the lead point for intussusception [14]. Our report of a jejunojejunal intussusception due to a heterotopic pancreas is exceptionally rare.

Successful management of intussusception in adults, regardless of the cause or location, will usually involve surgical resection of the lead point since, unlike pediatric intussusception, a pathological lesion is often found. Excision is particularly recommended given the risk for malignancy [4]. The radiological evaluation of suspected ectopic pancreatic tissue and its complications include oral contrast studies and CT (with and without intravenous contrast). Endoscopic ultrasound and an endoscopic ultrasound-guided fine-needle aspiration have been reported to aid in the diagnosis, depending on the location and accessibility of the lesion. However, none of the imaging modalities are diagnostic of a heterotopic pancreas, especially since the imaging findings can be seen with most bowel neoplasms. In addition to this, a smooth, broad-based intramural tumor with a central contrast-filled pit is the typical radiological appearance on barium studies [15]. Surgical resection is thus always needed to confirm the diagnosis and to provide further histological detail [16].

## Conclusions

In conclusion, the presence of heterotopic pancreas is rare and even rarer is the complication of small bowel intussusception from it. Pancreatic heterotopia is mostly discovered incidentally and for this reason, preoperative diagnosis is almost always impossible. Surgical resection is the most effective treatment, as the potential malignant transformation of the lesion should always be a consideration. Although an ectopic pancreas is still considered rare, it is highly recommended that it be considered as a differential diagnosis when evaluating cases of small bowel intussusception.
